# Comparing the Readability and Content Quality of Online Patient Education Materials and ChatGPT-Generated Patient Education Materials for Breast Cancer Surgery and Reconstruction

**DOI:** 10.1055/a-2794-9984

**Published:** 2026-03-27

**Authors:** Nikhil Sriram, Anitesh Bajaj, May Li, Tarifa Adam, Antoinette Nguyen, Jeewon Chon, Robert D. Galiano

**Affiliations:** 1 Division of Plastic and Reconstructive Surgery, Northwestern University Feinberg School of Medicine, Chicago, Illinois, United States; 2 Department of Surgery, University of Rochester School of Medicine and Dentistry, Rochester, Illinois, United States; 3 Department of Surgery, Loyola University Chicago Stritch School of Medicine, Maywood, Illinois, United States

**Keywords:** artificial intelligence, breast cancer surgery, breast reconstruction, patient education materials, readability, clinical practice and education, breast/trunk, breast reconstruction-oncoplastic surgery, hospital management

## Abstract

**Background:**

Patients undergoing breast cancer surgery and reconstruction seek information using online patient education materials (OPEMs). The National Institutes of Health (NIH) and American Medical Association (AMA) recommend a sixth-grade reading level for OPEMs. In recent years, Chat Generative Pre-Trained Transformer (ChatGPT), a large language model (LLM), has shown potential utility in patient education. This study compares the readability and content quality of OPEMs on breast cancer surgery and reconstruction with ChatGPT-generated materials.

**Methods:**

Google searches were conducted in January 2025 to identify relevant OPEMs for breast cancer surgery and reconstruction. For each search term, ChatGPT 4.0 was prompted to generate patient education guides using two approaches: (1) Standard prompting and (2) simplified prompting to align with NIH/AHA recommendations (“write the guide like I am in sixth grade”). Readability and content quality metrics were assessed.

**Results:**

Ninety-nine OPEMs and 60 ChatGPT responses (30 standard, 30 simplified) were analyzed. Median Flesch–Kincaid Grade Level (FKGL) was 10.8 for OPEMs, 10.0 for standard ChatGPT responses, and 5.8 for simplified ChatGPT responses. OPEMs and standard ChatGPT responses significantly exceeded NIH/AMA recommendations ( *p* < 0.001). Simplified ChatGPT responses aligned with the sixth-grade level and were significantly easier to read than OPEMs and standard ChatGPT responses ( *p* < 0.001). DISCERN scores did not significantly differ between OPEMs and standard/simplified ChatGPT responses.

**Conclusion:**

OPEMs on breast cancer surgery and reconstruction exceed recommended readability levels. ChatGPT, when prompted to simplify, produced materials consistent with NIH/AMA guidelines while maintaining content quality. Using ChatGPT for patient education may enhance accessibility and patient comprehension of health information.

## Introduction

Health literacy can be defined as an individual's ability to understand and act upon one's own health, including the ability to find, read, and understand written health information, communicate needs to health professionals, and act upon health instructions. 1 Patients with sufficient health literacy are able to better comprehend the causes, prognosis, and treatment of their illness; greater patient knowledge enables enhanced decision-making, increased patient engagement in care and satisfaction, and greater adherence to treatment. 1 2 Low health literacy has consistently been associated with poorer health outcomes, including more hospitalizations, poorer adherence to medication, and impaired ability to interpret labels and health messages. 3 In 2003, results from the National Assessment of Adult Literacy survey demonstrated that only 13% of Americans had “proficient” health literacy to understand prose, integrate documents, and employ quantitative information. 4 More recently, in 2023, results from the U.S. Program for the International Assessment of Adult Competencies suggested that 28% of adults performed at the lowest proficiency level in literacy. 5 As such, there is a need to maintain appropriate readability for patient education materials (PEMs). Specifically, the National Institutes of Health (NIH) and American Medical Association (AMA) have recommended that the readability of PEMs should be no greater than a sixth-grade reading level. 6 7 8

With the proliferation of the internet and search engines, online health information seeking behavior has become a global trend. 9 Prior studies have found that 8 out of 10 internet users have retrieved health information online, emphasizing its commonality; such information can impact health-related decision-making. 10 As such, online patient education materials (OPEMs) are now a common and highly accessible resource from which patients obtain health information. 9 Several studies have analyzed the quality and readability of OPEMs across a variety of medical specialties, including surgery, trauma, and radiology, all of which have consistently found that OPEMs tend to be of poor content quality and high reading level, often exceeding the NIH/AMA sixth-grade reading level recommendations. 5 6 11 Breast cancer surgery and reconstruction specifically are complex, often elective procedures where patients are faced with several choices and an abundance of information, often relying on online resources to aid in their understanding and decision-making process. While a wealth of OPEMs are available for breast cancer surgery and reconstruction, they have consistently demonstrated low readability, specifically advanced high-school levels and beyond. 12 13 14 15 The variability in readability and accuracy of online resources makes equitable, standardized patient education for breast cancer surgery and reconstruction challenging, as some patients may find resources inaccessible and fail to adequately comprehend presented information.

With the advent of generative artificial intelligence (AI), platforms such as Chat Generative Pre-Trained Transformer (ChatGPT) have become a new medium for health education. Recent work has explored the potential of these large language models (LLMs) in efficiently generating PEMs of greater quality and readability. One study comparing urologic OPEMs from the Urology Care Foundation (UCF) to ChatGPT-generated PEMs found that both sources failed to meet official readability standards. However, once queried to simplify the response with the prompt, “Explain it to me like I am in sixth grade,” the readability of ChatGPT-generated PEMs subsequently surpassed that of UCF. 16 Thus, while initial AI-generated OPEMs were not of significantly better readability, these results suggest that with effective prompting, LLMs may be used to improve readability.

The goal of the present study is to build upon prior work by comparing the readability and content quality of OPEMs on breast cancer surgery and reconstruction with those generated by ChatGPT, assessing the impact of prompt modulation on readability and quality.

## Methods

### Web Site Collection

A cross-sectional study was conducted to assess available OPEMs on breast cancer surgery and reconstruction, and PEMs generated by ChatGPT. To collect OPEMs for readability analysis, a Google search was conducted for relevant web sites. The following search terms were queried: “breast cancer surgery,” “mastectomy,” “lumpectomy,” “breast reconstruction,” “autologous reconstruction,” and “implant-based reconstruction.” These search terms were selected in collaboration with R.D.G. and were chosen to simulate potential terms that patients may utilize when conducting an online search regarding breast cancer surgery and/or breast reconstruction.

All Google searches were performed using the Google Chrome web browser. To minimize bias in search results, searches were performed in incognito mode, with location services deactivated, browser data cleared, and caching turned off. The initial 20 non-duplicate search results per search term were evaluated. Results were excluded if they did not include free, publicly available patient-facing material (i.e., academic journal articles, provider-facing materials, or product advertisement web sites). OPEMs were categorized into one of three web site types: Academic/Hospital (e.g., Cleveland Clinic), private practice, or online health reference (e.g., WebMD).

### ChatGPT Data Collection

Once all web sites had been collected, analyses were conducted using ChatGPT 4.0 (OpenAI, San Francisco, CA). The following prompt was passed through ChatGPT: “Generate a patient education guide for [search term].” To elicit simplified responses in-line with NIH/AMA recommendations, a second prompt was utilized: “Generate a patient education guide for [search term]. Write the guide like I am in sixth grade.” The search term in each prompt corresponded to one of the aforementioned search terms. Similar bias mitigation techniques were employed for ChatGPT analysis as were described for web site collection, such as queries performed in incognito mode, with location and cache disabled. In addition, memory was turned off when querying ChatGPT. Each prompt for each search term, standard and simplified, was queried five times to assess the degree of variability in response.

### Readability Analysis

Following the collection of web sites and ChatGPT-generated responses, a readability analysis was performed. Automated analysis was conducted using WebFX, a publicly available web site to evaluate readability. 17 WebFX contains several readability metrics, including grade-level indicators and statistics on the number of sentences, words, and complex words, all of which can be used to study the complexity of text. For this study, the following metrics were utilized: Flesch–Kincaid Grade Level (FKGL), Flesch–Kincaid Reading Ease Score (FKRE), Gunning Fog Score, Simple Measure of Gobbledygook (SMOG) Index, Coleman–Liau Index, and Automated Readability Index. These metrics are widely used measures of readability, commonly employed for health literacy research. 6 13 15 18 A lower FKGL, Gunning Fog score, SMOG Index, Coleman–Liau Index, and Automated Readability Index indicate a higher degree of readability, while a higher FKRE indicates a higher degree of readability. Prior studies have noted that the suggested readability score to align with recommendations is ≤6 for the FKGL, Gunning Fog score, SMOG Index, Coleman–Liau Index, and Automated Readability Index, and greater than 80.0 for the FKRE. 19 The free text on each web site was passed through to collect the readability metrics. Subsequently, the free text on each ChatGPT-generated material was passed through to collect the corresponding readability metrics. When assessing materials relative to the NIH/AMA sixth-grade readability recommendation, the FKGL was utilized.

### Content Quality Analysis

To study content quality for OPEMs and ChatGPT responses, the DISCERN tool was utilized. DISCERN is a validated instrument to study the quality of written health information and has previously been utilized to assess the quality of OPEMs as well as LLM-generated material. 11 19 20 21 DISCERN has a total of 16 questions, subdivided into three sections. Section #1 (Questions 1–8) appraises the information's reliability, Section #2 (Questions 9–15) evaluates the quality, and Section #3 is a single question (Question 16) to gauge overall quality. Each question is assigned a score on a scale of 1 to 5 (1 indicating no fulfillment of quality criteria and 5 indicating full quality criteria fulfillment). Thus, total scores can range from a minimum of 16 to a maximum of 80. Two reviewers independently completed DISCERN analysis for each OPEM and each ChatGPT response, and scores were subsequently averaged. DISCERN score quality interpretation is as follows: Excellent (63–80), good (51–62), medium (39–50), poor (27–38), or very poor quality (16–26). 11

### Statistical Analysis

Descriptive statistics were calculated for the various quantitative metrics for both OPEM and ChatGPT responses and were reported as medians with interquartile ranges (IQRs). Due to the nonparametric nature of the data, the readability metrics and DISCERN score between OPEMs, standard ChatGPT responses, and simplified ChatGPT responses were compared using the Kruskal–Wallis test followed by Dunn's post hoc test with Bonferroni correction for multiple comparisons. Wilcoxon signed-rank tests were used to individually compare the FKGL of OPEMs, standard ChatGPT responses, and simplified ChatGPT responses to the NIH/AMA sixth-grade readability recommendations. The significance level for all statistical tests was set at <0.05. All statistical analysis was performed in R (4.4.3).

## Results

A total of 120 web sites were analyzed across the six search terms. Ninety-nine web sites met the inclusion criteria and were included in subsequent analysis. Ninety (91%) were academic/hospital web sites, five (5%) were online health reference sites, and four (4%) were private practice web sites. A total of 60 ChatGPT responses were generated. There were 30 standard responses generated, 5 responses generated per keyword, while the remaining 30 responses generated included prompting to simplify to a sixth-grade level, with 5 responses generated per keyword. Included OPEMs, ChatGPT responses, and collected readability and quality scores are available in a public repository. 22

### Readability

**Fig. 1:**
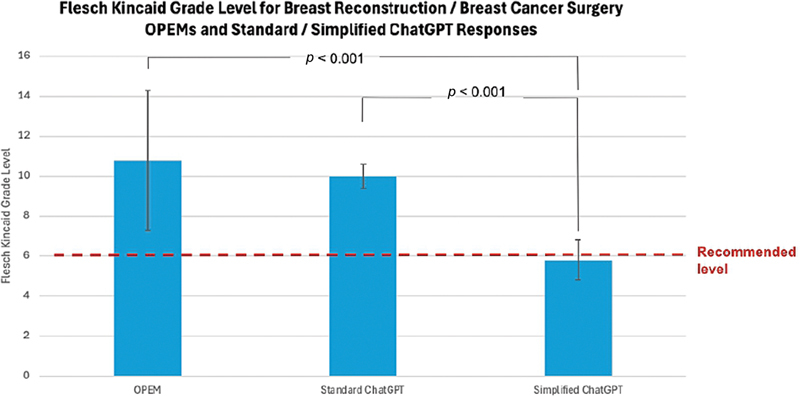
Flesch–Kincaid Grade Level by modality. ChatGPT, Chat Generative Pre-Trained Transformer; OPEM, online patient education material.

The median FKGL of queried OPEMs was 10.8 (9.4–12.8). The median FKGL of standard ChatGPT responses was 10.0 (9.8–10.4). The median FKGL of simplified ChatGPT responses was 5.8 (5.3–6.4; Fig. 1 and Table 1 ). There was a significant difference in FKGL across the three patient education modalities ( *p* < 0.001). Pairwise testing showed that the median FKGL of simplified ChatGPT responses was significantly lower than that of OPEMs ( *p* < 0.001) and of standard ChatGPT responses ( *p* < 0.001), indicating an increased ease of readability in simplified ChatGPT responses ( Fig. 1 and Table 1 ). OPEMs had a median FKRE score of 51.2 (43.8–58.7), considered “fairly difficult” to read. Standard ChatGPT responses had a median FKRE score of 42.9 (40.4–44.3), considered “difficult” to read. Simplified ChatGPT responses had a median FKRE score of 74.3 (71.6–77.1), interpreted as “fairly easy” to read ( Table 1 ). Full results across all readability metrics are shown in Table 1 .

**Table 1:** Readability metrics across online patient education materials, standard ChatGPT responses, and simplified ChatGPT responses

Readability metric	OPEMs	Standard ChatGPT	Simplified ChatGPT	OPEM-Standard ChatGPT *p* -value	OPEM-Simplified ChatGPT *p* -value	Standard ChatGPT-Simplified ChatGPT *p* -value
Flesch–Kincaid Reading Grade Level	10.8 [9.4–12.8]	10.0 [9.8–10.4]	5.8 [5.4–6.4]	0.264	<0.001 a	<0.001 a
Flesch–Kincaid Reading Ease Score	51.2 [43.8–58.7]	42.9 [40.4–44.3]	74.3 [71.6–77.1]	<0.001 a	<0.001 a	<0.001 a
Gunning Fog Score	13.9 [12.2–15.7]	11.8 [11.4–12.5]	7.9 [7.4–8.3]	0.137	<0.001 a	<0.001 a
SMOG Index	10.5 [9.1–11.5]	9.5 [9.2–9.6]	6.5 [6.2–6.8]	0.137	<0.001 a	<0.001 a
Coleman–Liau Index	13.7 [12.3–14.4]	16.7 [16.0–17.5]	11.1 [10.4–11.6]	<0.001 a	<0.001 a	<0.001 a
Automated Readability Index	11.9 [9.6–13.8]	10.1 [9.5–10.6]	5.8 [5.3–6.4]	0.110	<0.001 a	<0.001 a

Abbreviations: ChatGPT, Chat Generative Pre-Trained Transformer; OPEM, online patient education material; SMOG Index, Simple Measure of Gobbledygook Index.

Results were reported as median [interquartile range]. A higher Flesch–Kincaid Reading Ease Score indicates increased ease of readability; a lower value for all other metrics indicates increased ease of readability.

a Indicates *p* -value <0.05.

This table shows the median value for six readability metrics assessed across three patient education modalities: Online patient education materials, standard ChatGPT responses, and simplified ChatGPT responses. Across all readability metrics studied, simplified ChatGPT responses show a significant difference compared to online patient education materials and standard ChatGPT responses, indicating increased ease of readability.

Throughout the six readability metrics studied—FKGL, FKRE, Gunning Fog Score, SMOG Index, Coleman–Liau Index, and Automated Readability Index—there was a significant difference between OPEMs and simplified ChatGPT responses as well as between standard and simplified ChatGPT responses ( Table 1 ). OPEMs and standard ChatGPT responses showed significant differences on two readability metrics: FKRE and Coleman–Liau Index ( Table 1 ). The median FKGL of simplified ChatGPT responses was at par with the NIH/AMA sixth-grade recommendations ( *p* = 0.19), while the median FKGL of OPEMs and standard ChatGPT responses were both significantly greater than the NIH/AMA sixth-grade recommendations ( *p* < 0.001; Fig. 1 ). The FKGL IQR, a measure of variability, for standard and simplified ChatGPT responses was 0.6 and 1.0, respectively, while the FKGL IQR for OPEMs was 3.4 ( Fig. 1 and Table 1 ). The IQR of OPEMs was larger than that of standard and simplified ChatGPT responses for all readability metrics assessed ( Table 1 ). Analysis segmented by search term showed similar results, with a significant difference observed in median FKGL between the three patient education modalities for every search term studied; simplified ChatGPT responses had the lowest FKGL across all search terms, indicative of increased ease of readability ( Table 2 ).

**Table 2:** Flesch–Kincaid Grade Level by modality and by search term

Search term	OPEM FKGL	Standard ChatGPT FKGL	Simplified ChatGPT FKGL	*p* -Value
Breast cancer surgery	10.8 [9.7–12.7]	9.8 [9.1–9.8]	5.5 [5.2–5.8]	0.0012 a
Mastectomy	9.9 [8.5–11.8]	10.3 [10.2–10.9]	6.4 [6.1–6.5]	0.0026 a
Lumpectomy	10.3 [8.6–12.3]	9.8 [9.8–10.2]	5.4 [5.3–5.8]	0.0024 a
Breast reconstruction	11.5 [8.8–12.8]	10.2 [9.4–11.2]	5.8 [5.3–6.2]	0.0021 a
Autologous reconstruction	10.5 [9.9–12.4]	10.4 [10.3–10.8]	5.9 [5.8–6.7]	0.0049 a
Implant-based reconstruction	13.0 [11.2–14.0]	9.9 [9.8–10]	6.0 [5.7–6.4]	<0.001 a

Abbreviations: ChatGPT, Chat Generative Pre-Trained Transformer; FKGL, Flesch–Kincaid Grade Level; OPEM, online patient education material.

Results were reported as median [interquartile range]. Lower Flesch–Kincaid Grade Level indicates increased ease of readability.

a Indicates *p* -value <0.05.

This table shows the median FKGL segmented by search term assessed across three patient education modalities: OPEMs, standard ChatGPT responses, and simplified ChatGPT responses. Across all search terms, simplified ChatGPT responses show the lowest FKGL compared to online patient education materials and standard ChatGPT responses, indicating increased ease of readability.

### Content Quality

**Fig. 2:**
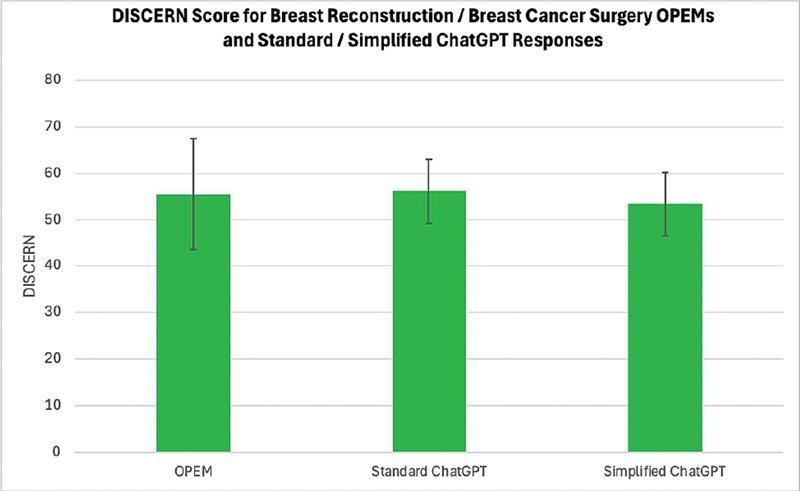
DISCERN score by modality. ChatGPT, Chat Generative Pre-Trained Transformer; OPEM, online patient education material.

The median DISCERN scores of OPEMs (55.5, IQR 50.3–62.3), standard ChatGPT responses (56.2, IQR 52.6–59.5), and simplified ChatGPT responses (53.5, IQR 49.8–56.5), all fell within the range considered “good” quality ( Fig. 2 ). There was no significant difference in median DISCERN score between the three patient education modalities ( *p* = 0.083). Analysis segmented by search term showed similar results, with no significant difference observed in median DISCERN score between the three patient education modalities for every search term studied ( Table 3 ). Across all three modalities, the lowest DISCERN score was seen with the lumpectomy search term ( Table 3 ). The DISCERN IQR for standard and simplified ChatGPT responses was 6.9 and 6.7, respectively, while the DISCERN IQR for OPEMs was 12.0 ( Fig. 2 ).

**Table 3:** DISCERN by modality and by search term

Search term	OPEM DISCERN	Standard ChatGPT DISCERN	Simplified ChatGPT DISCERN	*p* -Value
Breast cancer surgery	55.5 [51.3–59.8]	56.0 [51.5–56.0]	51.0 [50.5–56.5]	0.630
Mastectomy	55.0 [51.0–59.5]	56.0 [54.5–56.0]	51.0 [48.0–53.0]	0.370
Lumpectomy	48.5 [39.5–55.4]	53.0 [52.0–57.0]	49.5 [48.0–51.0]	0.193
Breast reconstruction	63.5 [58.3–66.3]	59.5 [57.5–62.0]	55.0 [53.5–56.5]	0.067
Autologous reconstruction	58.0 [52.5–63.3]	60.5 [56.5–60.5]	56.0 [53.0–57.5]	0.466
Implant-based reconstruction	54.8 [49.8–58.4]	56.5 [55.5–58.0]	56.0 [55.0–57.0]	0.725

Abbreviations: ChatGPT, Chat Generative Pre-Trained Transformer; OPEM, online patient education material.

Results reported as median [interquartile range].

Table 3 shows the median DISCERN score segmented by search term assessed across three patient education modalities: OPEMs, standard ChatGPT responses, and simplified ChatGPT responses. Across all search terms, no significant difference was observed in DISCERN score across the three modalities.

## Discussion

This study sought to analyze the readability and content quality of OPEMs in breast cancer surgery and reconstruction and assess the ability for ChatGPT to generate OPEMs at a guideline-directed reading level. Our findings indicated that current OPEMs significantly exceed the NIH/AMA-recommended reading levels. ChatGPT, with no prompting for simplification, showed a relatively similar level of readability compared with OPEMs. When prompted to simplify the material, ChatGPT significantly improved readability to align with recommendations. In addition, no difference in content quality was observed between the three patient education modalities as measured by the DISCERN instrument.

OPEMs are vital sources of information for patients to better understand their health concerns and are increasingly leveraged by health care consumers to take ownership of their health. A previous survey study found that patients who engaged with online health information had an increased likelihood of following physician recommendations and asking more questions during clinic visits. 23 With the democratization of medical information, it is important to understand the factors that may lead to impaired comprehension. Providers of this online information should ensure a baseline level of readability, quality, and reliability, as inaccurate or incomprehensible health information can have detrimental consequences for patients. 24 This is especially important in the setting of sensitive procedures like breast cancer surgery and reconstruction, where patient understanding of the pros and cons of the possible options is paramount to appropriate decision-making. Materials written with complexity can impair the delivery of care, as patients with limited health literacy may not be able to engage in shared decision-making discussions, which are crucial in the setting of breast surgery and reconstruction. 25

Our analysis indicates a mismatch between the median reading level of OPEMs and national recommendations, highlighting a key area for improvement in patient-facing communication. Our findings show that when prompted, ChatGPT is able to produce content that aligns with sixth-grade readability standards and shows less variability in response compared with OPEMs. This suggests a potential pathway to address health literacy gaps, particularly in contexts where the decision-making burden on patients is high, like breast cancer surgery and reconstruction. The consistent reduction in complexity across readability metrics underscores the importance of prompt design, highlighting that how LLMs are queried to generate information is fundamental in the generation of desired output. Prior studies have shown similar results, with LLMs significantly improving readability when prompted. 26 27 28 However, in a 2023 study, Hung et al used ChatGPT 3.5, the predecessor to the model used in this study, to generate PEMs for breast reconstruction and found that ChatGPT-generated responses were not able to align readability level with standards. 29 In our study, ChatGPT 4.0, when prompted, produced health information that aligned with readability standards. Prior literature has similarly shown ChatGPT 4.0 to be more effective than ChatGPT 3.5 in simplifying readability level when prompted, indicating that advancements in ChatGPT models, alongside specified prompting, may also be an important factor in the degree of simplification the LLM is capable of. 28 30 Improvement in OPEMs for plastic surgery continues to be needed, as demonstrated in this study and prior literature. Wirth et al, in 2025, noted some improvements in the readability of PEMs from the American Society of Plastic Surgeons (ASPS) and The Aesthetic Society (AS) web sites over the past decade; however, these web sites were still written at a 10th-grade reading level or above, significantly higher than recommendations. 31 Though LLMs offer an innovative approach to modulate readability, several strategies have also previously been described to improve the accessibility and readability of OPEMs, such as utilizing short sentences and a conversational tone, presenting information through examples and analogies, limiting medical jargon, offering basic and advanced versions to accommodate for differences in literacy, providing abundant supplemental material such as pictures and videos, and encouraging members of national societies to share links to accessible materials via social media. 23 31 32 Moreover, online web mediums and platforms such as YouTube, which are currently at the forefront of health information gathering among patients, may also integrate AI and LLM tools to facilitate the dissemination of information to their audience. 10 19 33

Moreover, our findings indicate that reducing linguistic complexity did not degrade the quality of content as measured by the DISCERN instrument. Simplified ChatGPT responses retained key informational elements and scored comparably to both standard responses and traditional OPEMs. In addition, ChatGPT responses showed less variability in content quality compared with OPEMs. Prior literature applying the DISCERN score to LLM-generated material has shown similar results, with LLM-generated material typically having “good” quality or quality comparable to that of OPEMs. 21 34 35 Similar to readability, Hung et al previously found ChatGPT 3.5 to have moderate accuracy, with some information errors. 29 In our study, ChatGPT 4.0 responses, both standard and simplified, were found to have “good” quality. Prior literature has similarly identified ChatGPT 4.0 to have greater response quality and DISCERN scores than ChatGPT 3.5. 36 A recent study from Marcaccini et al found ChatGPT 4.0 to have strong performance across four criteria—relevance, accuracy, clarity, and empathy—when answering questions related to breast reconstruction, mastopexy, breast augmentation, and breast reduction. 37 Thus, newer LLM models, including ChatGPT 4.0, may serve as useful mediums to deliver health information to patients as an adjunct to information received from providers.

A risk of widespread use of LLMs in a clinical setting is that the training datasets are always changing, making the information they use to generate outputs dynamic. To address this, the use of source-grounded LLMs may be more trustworthy in sensitive health care settings where LLMs are linked to verifiable information sources, which helps minimize any hallucinations. 38 An application of this would be to ground an LLM in plastic surgery organizational educational guides and subsequently tailor the output to a guideline-directed reading level, as our study demonstrated, to produce comprehensible, evidence-based information that supplements education delivered by providers. Nonetheless, other challenges of using LLMs, such as ChatGPT, for medical education remain, including the risk of false or hallucinated content, lack of source citation, and ethical considerations regarding patient privacy, trust, and communication. 39

The present study has multiple limitations. First, ChatGPT and other LLMs create outputs based on the data they are trained on, which dynamically change over time. Therefore, the findings from this cross-sectional study may be impacted by the evolution of LLMs over time. Other publicly available LLMs, such as Google Gemini and Claude, have also been studied as patient education tools, showing comparable performance to ChatGPT. 19 40 These LLMs should be explored in future literature within breast surgery. In addition, this search is limited by using six search terms, which may not fully represent the full spectrum of search terms that patients may use when searching online sources related to breast cancer surgery and breast reconstruction. Moreover, only one search engine was used, which could impact the OPEMs collected. A single tool, WebFX, was utilized to calculate readability metrics, which may introduce some measurement error, as estimates can vary between different tools. 20 Furthermore, OPEMs may undergo revisions and updates over time, which can alter readability and quality metrics. Finally, the validity of the LLM patient education materials generated in this study has not yet been tested in real patients, which may limit the direct clinical applicability of the findings. Future studies should aim to survey patient comprehension of LLM-generated health information versus OPEMs in breast cancer and reconstructive surgery. Nonetheless, the present study offers an in-depth assessment of ChatGPT 4.0 for breast cancer surgery and reconstruction and presents a novel assessment of the impact of prompt variation on and variability between ChatGPT responses. This study is also the first to apply the DISCERN metric to LLM-generated material for breast cancer surgery and reconstruction.

### Conclusion

The findings of this study demonstrate that ChatGPT, when prompted, is an effective medium to improve the readability of OPEMs with respect to national guidelines for breast cancer surgery and breast reconstruction while maintaining content quality. As AI continues to evolve, it may have utility in improving the accessibility of health information for patients within plastic surgery.
